# Deciphering *Clostridium tyrobutyricum* Metabolism Based on the Whole-Genome Sequence and Proteome Analyses

**DOI:** 10.1128/mBio.00743-16

**Published:** 2016-06-14

**Authors:** Joungmin Lee, Yu-Sin Jang, Mee-Jung Han, Jin Young Kim, Sang Yup Lee

**Affiliations:** a Metabolic and Biomolecular Engineering National Research Laboratory, Department of Chemical and Biomolecular Engineering (BK21 Plus Program), KAIST, Yuseong-gu, Daejeon, South Korea; b Department of Biomolecular and Chemical Engineering, Dongyang University, Yeongju, Gyeongbuk, South Korea; c Mass Spectrometry Research Center, Korea Basic Science Institute, Cheongju, Chungbuk, South Korea; d Bioinformatics Research Center, KAIST, Daejeon, South Korea; e BioProcess Engineering Research Center, Center for Systems and Synthetic Biotechnology, KAIST, Daejeon, South Korea; f Institute for the BioCentury, KAIST, Daejeon, South Korea

## Abstract

*Clostridium tyrobutyricum* is a Gram-positive anaerobic bacterium that efficiently produces butyric acid and is considered a promising host for anaerobic production of bulk chemicals. Due to limited knowledge on the genetic and metabolic characteristics of this strain, however, little progress has been made in metabolic engineering of this strain. Here we report the complete genome sequence of *C. tyrobutyricum* KCTC 5387 (ATCC 25755), which consists of a 3.07-Mbp chromosome and a 63-kbp plasmid. The results of genomic analyses suggested that *C. tyrobutyricum* produces butyrate from butyryl-coenzyme A (butyryl-CoA) through acetate reassimilation by CoA transferase, differently from *Clostridium acetobutylicum*, which uses the phosphotransbutyrylase-butyrate kinase pathway; this was validated by reverse transcription-PCR (RT-PCR) of related genes, protein expression levels, *in vitro* CoA transferase assay, and fed-batch fermentation. In addition, the changes in protein expression levels during the course of batch fermentations on glucose were examined by shotgun proteomics. Unlike *C. acetobutylicum*, the expression levels of proteins involved in glycolytic and fermentative pathways in *C. tyrobutyricum* did not decrease even at the stationary phase. Proteins related to energy conservation mechanisms, including Rnf complex, NfnAB, and pyruvate-phosphate dikinase that are absent in *C. acetobutylicum*, were identified. Such features explain why this organism can produce butyric acid to a much higher titer and better tolerate toxic metabolites. This study presenting the complete genome sequence, global protein expression profiles, and genome-based metabolic characteristics during the batch fermentation of *C. tyrobutyricum* will be valuable in designing strategies for metabolic engineering of this strain*.*

## IMPORTANCE

Bio-based production of chemicals from renewable biomass has become increasingly important due to our concerns on climate change and other environmental problems. *C. tyrobutyricum* has been used for efficient butyric acid production. In order to further increase the performance and expand the capabilities of this strain toward production of other chemicals, metabolic engineering needs to be performed. For this, better understanding on the metabolic and physiological characteristics of this bacterium at the genome level is needed. This work reporting the results of complete genomic and proteomic analyses together with new insights on butyric acid biosynthetic pathway and energy conservation will allow development of strategies for metabolic engineering of *C. tyrobutyricum* for the bio-based production of various chemicals in addition to butyric acid.

## INTRODUCTION

As agreed at the 21st Conference of the Parties (COP-21) meeting in Paris, France, on urgent action to be taken for addressing climate change, it is necessary to reduce our dependence on fossil resources and move toward bio-based production of chemicals, fuels, and materials from renewable resources. Among the chemicals that can be produced by microorganisms, butyric acid is an important industrial chemical that has been used for the production of short-chain esters for perfume and food flavoring agents (1). In addition, butyric acid can be used to produce various pharmaceuticals such as vasoconstrictor drugs, anticancer agents, and antioxidants (2). 

*Clostridium tyrobutyricum* is a Gram-positive, strictly anaerobic bacterium that most efficiently produces butyric acid. This organism has received much attention in the dairy industry because contamination by *C. tyrobutyricum* spores in cheese fermentation processes often causes the late-blowing defect, resulting in inferior cheese quality mainly due to the undesirable flavor of butyric acid (3–5). *C. tyrobutyricum* can be employed as a great microbial cell factory for the production of various chemicals due to several advantages including its ability to grow on a minimal medium, high metabolic flux toward butyric acid, and tolerance to the product at high concentrations (6). Despite such potential, *C. tyrobutyricum* has received less attention as an industrial host strain compared to other clostridial species, mainly due to our limited understanding on its genotypic and metabolic characteristics at the genome level or scale. Most studies of this bacterium so far have been focused on the optimization of fermentation processes (7–10). To date, only a few metabolic genes of *C. tyrobutyricum* have been knocked out (11–13). It has been reported that deletion of the *pta* gene encoding phosphotransacetylase (PTA) resulted in reduction of PTA activity up to 60%, and consequently increased butyric acid titer by 68% (12). Deletion of the *ack* gene encoding acetate kinase (AK) led to a similar result, increasing the butyric acid titer and yield (14). More recently, deletion of the *ptb* gene encoding phosphotransbutyrylase (PTB) has been demonstrated, but the butyric acid yield of the *ptb* mutant was similar to that of the wild-type strain despite its reduced PTB activity (13).

In addition to butyric acid, *C. tyrobutyricum* has great potential to be employed for the production of other chemicals. A recent study on the metabolic engineering of *C. tyrobutyricum* suggested that overexpression of *C. acetobutylicum adhE2* in an *ack*-deleted *C. tyrobutyricum* strain resulted in production of 10 g liter^−1^ of 1-butanol from glucose (15). It was demonstrated that *C. tyrobutyricum* could tolerate 1-butanol at the initial concentration of 15 g liter^−1^, at which a native 1-butanol producer *Clostridium beijerinckii* ATCC 55025 was not able to grow (15). More recently, optimizing the expression of *Clostridium acetobutylicum adhE2* in *C. tyrobutyricum* led to the production of 20 g liter^−1^ of 1-butanol from mannitol without manipulating the competing pathways (16); this 1-butanol concentration is higher than those obtained by native 1-butanol producers (17, 18). Even though mannitol is a more-reduced substrate than glucose, this result suggests that further improvement could be achieved in *C. tyrobutyricum* through systems metabolic engineering (19, 20). More recently, a draft genome sequence of *C. tyrobutyricum* has been reported (21, 22). Based on the draft genome sequence, proteomic studies of the wild-type and mutant strains of *C. tyrobutyricum* have been performed (23). However, the lack of complete genome sequence and transcriptomic and/or proteomic studies during the course of fermentation has limited our understanding of the physiological characteristics of *C. tyrobutyricum* for subsequent metabolic engineering at the systems level.

In this study, we report the complete genome sequence of *C. tyrobutyricum* KCTC 5387 (ATCC 25755) and its proteome profiles during the course of batch fermentation. Strikingly, the complete genome sequence suggested that this bacterium does not use PTB and butyrate kinase (BK) for butyric acid production; instead, a coenzyme A (CoA) transferase (CoAT) mediates butyric acid production from butyryl-CoA by reassimilation of acetic acid, which is produced by PTA and AK. Also, several energy conservation mechanisms in *C. tyrobutyricum*, which are responsible for its capability to efficiently produce butyric acid and tolerate toxic products, were identified by proteomic analyses during the course of fermentation.

## RESULTS

### Features of the complete *C. tyrobutyricum* genome.

**FIG 1:**
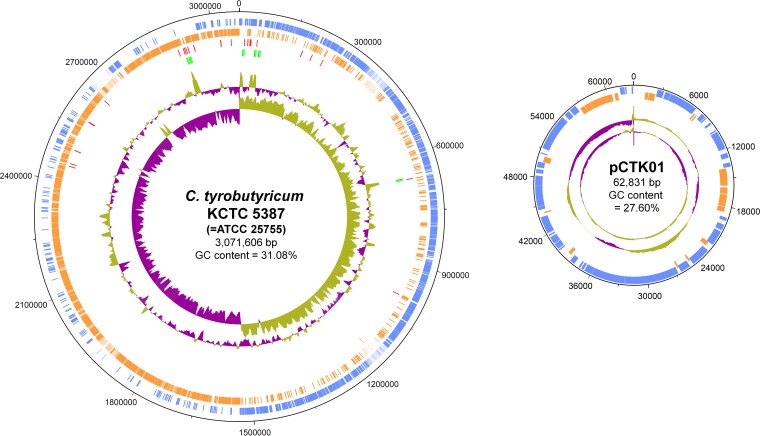
Genome atlas view of the *C. tyrobutyricum* KCTC 5387 (ATCC 25755) chromosome and the pCTK01 plasmid. The outermost ring shows the coordinates of the chromosome or plasmid. The coding sequences in the forward and reverse strands are colored blue and orange, respectively. tRNAs and rRNAs are indicated by red and green, respectively. The fourth and fifth rings represent the deviations of GC content and GC skew, i.e., (G − C)/(G + C), from their average values, respectively; purple, negative deviation (below average); green, positive deviation (above average).

The general features of the *C. tyrobutyricum* genome are summarized in Table 1. The important statistics are generally in agreement with the draft sequences reported recently (21, 22). The genome of *C. tyrobutyricum* KCTC 5387 contains one circular 3.07-Mbp chromosome and one circular 63-kbp plasmid (designated pCTK01; Fig. 1A). The strand bias of the chromosomal genes is one important characteristic in Firmicutes (24), and the origin and terminus of replication of the chromosome are located at nearly 0° and 180°, respectively; about 80% of the chromosomal genes are carried on the leading strand. The locations of the chromosomal replication origin and terminus are consistent with those predicted by GC skew analysis (Fig. 1). There are two 52-kbp duplicated regions at 116° and 122° on the chromosome; the majority of the genes in these clusters are those with unknown functions, and some of them encode phage-related proteins.

**TABLE 1:** General features of *C. tyrobutyricum* genome and comparison with other closely related clostridial species

Parameter	Value^a^ for parameter in:		
	*C. tyrobutyricum* KCTC 5387 (ATCC 25755)	*C. kluyveri* DSM 555	*C. ljungdahlii* DSM 13528
Chromosome size (bp)	3,071,606	3,964,618	4,630,065
Plasmid size (bp)	62,831	59,182	NA
GC content (%)	31.0	32.0	23.0
No. of total genes	3,220 (78)	3,994 (75)	4,283
ORFs	3,138 (78)	3,913 (75)	4,184
rRNAs	19	20	27
tRNAs	63	61	72

a he numbers in parentheses are the number of genes on the plasmid. NA, not available.

Like pCKL555A in *Clostridium kluyveri* (25), a species taxonomically close to *C. tyrobutyricum*, the genes on the pCTK01 plasmid have no clearly known functions, except for those encoding a DNA topoisomerase (CTK_P00130), a sigma factor (CTK_P00210), an arsenite transporter (CTK_P00580-610), and those encoding putative proteins for conjugal plasmid transfer (CTK_P00290-410). Plasmid pCTK01 shows neither the strand bias of the genes nor a clear transition of GC skew, suggesting that this plasmid might replicate via rolling-circle mechanism rather than theta-type mechanism (26). However, we could not find those known genes in pCTK01 for its replication, and thus, further studies are needed to understand how this plasmid replicates.

The genes in *C. tyrobutyricum* were functionally annotated using the Kyoto Encyclopedia of Genes and Genomes (KEGG) automatic annotation server (27), and the annotation results were compared with those for *C. kluyveri* and *Clostridium ljungdahlii*. Among the 3,138 protein-coding genes, 49.4% of them (1,552 genes) could be assigned with KEGG Orthology (KO) numbers. This result is slightly better than those of *C. kluyveri* (42.2%) and *C. ljungdahlii* (45.7%). Despite its smaller genome size, the number of KO categories assigned to *C. tyrobutyricum* genes was 1,257, which was comparable to those in *C. kluyveri* (1,248 KOs) and *C. ljungdahlii* (1,370 KOs).

### Shotgun proteomic analyses of *C. tyrobutyricum*.

**FIG 2:**
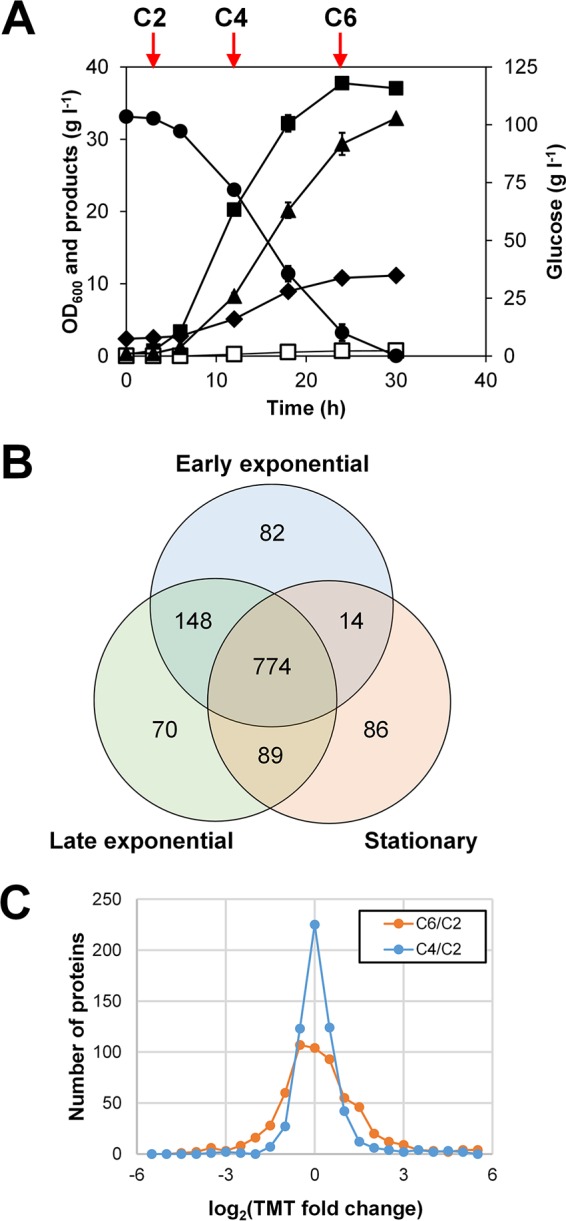
Summary of the *C. tyrobutyricum* shotgun proteomics. (A) Batch fermentation profiles used for the sample preparation. Symbols: closed circles, glucose; closed squares, OD_600_; closed diamonds, acetic acid; closed triangles, butyric acid; open squares, ethanol. (B) A Venn diagram of proteins identified at each sampling point for label-free shotgun proteomic data. The number of proteins is shown in each area. (C) Histograms that present relative protein fold changes at C4 and C6 compared to C2 based on TMT-tagged proteome samples.

In order to better understand metabolic characteristics of *C. tyrobutyricum*, protein expression profiles were examined during batch fermentation on glucose. Samples from duplicated batch fermentations of *C. tyrobutyricum* were subjected to shotgun proteomic analyses (Fig. 2A). About 100 g liter^−1^ of glucose was completely consumed within 30 h, and the maximum optical density at 600 nm (OD_600_) reached was about 38. The major fermentation products were acetic acid (11.1 g liter^−1^) and butyric acid (32.9 g liter^−1^), and larger or higher-carbon-numbered (>C_5_) fatty acids were not produced. While the butyric acid yield (0.32 g g^−1^ glucose) was similar to that reported in a previous study (23), the acetic acid yield was slightly higher in our result (0.11 versus 0.074 g g^−1^), possibly due to the different composition of the culture media. As expected from its genome sequence, acetone and butanol were not detected throughout the fermentation, but small amounts of formic acid (3.1 g liter^−1^) and ethanol (0.7 g liter^−1^) were produced.

Samples for proteome profiling were taken at early exponential (C2), late exponential (C4), and stationary (C6) phases (Fig. 2A). Proteins isolated from two independent samples at the same phase were mixed and fragmented using trypsin. The resulting peptide fragments were analyzed by high-performance liquid chromatography coupled to tandem mass spectrometry (HPLC-MS/MS). All unique peptide sequences identified were compared to the predicted amino acid sequences of *C. tyrobutyricum* genes. Estimated false-positive rates of peptides were 0.3% for C2, 0.7% for C4, and 1.0% for C6. A total of 1,263 proteins were identified during the course of *C. tyrobutyricum* fermentation, and of these proteins, 774 proteins were identified at all phases (Fig. 2B; see Data Set S1 in the supplemental material). The numbers of identified proteins at C2, C4, and C6 were 1,046, 1,082, and 963, respectively, which were categorized based on KO assignment in Table 2.

**TABLE 2:** *C. tyrobutyricum* proteins identified by label-free shotgun proteome during the course of batch fermentation

Functional category	No. of genes assigned	No. of proteins identified				
		C2	C4	C6	All phases	At least one phase
Total		1,018	1,081	963	774	1,263
Carbohydrate metabolism	168	79	94	90	74	96
Energy metabolism	111	60	58	55	52	63
Lipid metabolism	51	22	23	22	20	24
Nucleotide metabolism	82	65	59	58	53	69
Amino acid metabolism	155	112	115	107	97	124
Metabolism of other amino acids	34	22	23	21	18	26
Glycan biosynthesis and metabolism	20	11	12	10	10	12
Metabolism of cofactors and vitamins	110	59	68	60	42	79
Metabolism of terpenoids and polyketides	30	17	20	18	16	20
Biosynthesis of other secondary metabolites	16	12	12	11	11	12
Xenobiotic biodegradation and metabolism	38	14	11	13	10	16
Transcription	4	4	4	4	4	4
Translation	83	74	73	72	71	76
Folding, sorting, and degradation	38	24	23	25	20	28
Replication and repair	46	23	24	19	17	26
Membrane transport	107	32	37	34	30	38
Signal transduction	55	30	33	32	29	33
Cell motility	44	32	36	32	26	38
Cell growth and death	12	9	9	8	7	10
Not assigned	1600	257	300	246	168	375

From the label-free proteome data obtained, the relative abundance of the proteins were calculated using the normalized spectral abundance factors (NSAF; Data Set S1). Compared to C2, about 85% and 66% of proteins at C4 and C6, respectively, showed changes in their expression levels within twofold. Highly abundant proteins in all phases included 50S ribosomal protein L7/L12 (CTK_28970), 60-kDa chaperonin GroEL (CTK_C26260), elongation factor Tu (CTK_C28900 and CTK_C29040), thiolase (CTK_01450), and 3-hydroxybutyryl-CoA dehydrogenase (CTK_26330). These results are similar to those obtained from the shotgun proteomic analyses of *C. acetobutylicum* (28). One exceptional case was flavodoxin (CTK_C21890), which was only the 465th most abundant at C2, but the most abundant at C4 and the 5th most abundant at C6.

For more accurate determination of protein fold changes between the samples, we additionally performed relative quantification of the peptides labeled by Tandem Mass Tag (TMT) isobaric tags in duplicates for each sampling point (Fig. 2C; see Data Set S2 in the supplemental material). In this case, 587 proteins were identified. Proteins with significant fold changes (*P* value of 0.05) are shown in Table 3. Significantly upregulated genes include the genes involved in flagellar formation, iron transport, cofactor biosynthesis, stress response, and sporulation, whereas downregulated genes include genes encoding a transcription elongation factor, an ABC transporter, enzymes involved in fructose metabolism, and carbon monoxide dehydrogenase.

**TABLE 3:** Proteins with significant changes identified by TMT-labeled shotgun proteome

Protein no.	Locus	Description	Spectral count	C4/C2 ratio^a^	C6/C2 ratio^a^
1	CTK_C14560	Predicted ABC transporter with duplicated ATPase domain	3	−0.99	**−4.67**
2	CTK_C29270	Transcription elongation factor GreA	2	−0.65	**−3.90**
3	CTK_C06320	Fructose-specific PTS (phosphotransferase system) system enzyme IIABC component	4	**−3.16**	−3.70
4	CTK_C06300	Predicted transcriptional regulator, DeoR family	2	**−3.23**	−3.63
5	CTK_C06310	1-Phosphofructokinase	5	**−3.27**	−3.33
6	CTK_C21760	Anaerobic-type carbon monoxide dehydrogenase	4	**−2.65**	−3.09
7	CTK_C22200	Biotin synthase	3	**4.51**	1.48
8	CTK_C21480	FlgK	11	**2.12**	1.65
9	CTK_C20110	FlgG1	4	**2.29**	2.19
10	CTK_C20360	FlaB	5	**2.16**	2.26
11	CTK_C29600	Predicted membrane protein	5	**2.74**	2.55
12	CTK_C11790	Cadmium-, zinc-, and cobalt-transporting ATPase	9	**3.34**	2.62
13	CTK_C21790	Pyridoxal biosynthesis lyase PdxS	8	**3.52**	2.84
14	CTK_C21800	Glutamine amidotransferase subunit PdxT	3	**4.09**	2.84
15	CTK_C09160	l-Aspartate oxidase	3	**4.56**	3.19
16	CTK_C21410	Flagellar capping protein	7	**2.51**	3.28
17	CTK_C21890	Flavodoxin	140	**3.07**	3.36
18	CTK_C26570	Universal stress protein UspA-like protein	32	1.61	**3.91**
19	CTK_C27410	Adenosylmethionine-8-amino-7-oxononanoate transaminase	6	**3.30**	**3.92**
20	CTK_C01140	Hypothetical protein	4	−0.25	**3.99**
21	CTK_C01170	Hypothetical protein	21	−0.32	**4.38**
22	CTK_C16160	Ferrous iron transport protein B homolog	8	**3.72**	**4.70**
23	CTK_C08840	Anti-sigma F factor antagonist	3	**2.73**	**4.78**
24	CTK_C16180	FeoA-related protein	21	**4.67**	**4.96**
25	CTK_C18500	Stage IV sporulation protein A	7	−0.20	**4.98**
26	CTK_C09150	Quinolinate synthase	15	**4.94**	**5.06**
27	CTK_C14720	Putative uncharacterized protein	4	**3.96**	**5.32**
28	CTK_C12660	Putative glycosyltransferase, group I	4	**2.86**	**5.33**
29	CTK_C00980	Hypothetical protein	10	**5.16**	**5.43**
30	CTK_C19550	Predicted stage III sporulation protein AH	3	−0.12	**5.44**
31	CTK_C27380	Predicted serine kinase	3	−0.43	**5.57**
32	CTK_C30350	18-kDa heat shock protein	3	0.82	6.12

a The ratios for proteins with significant fold changes (*P* value of 0.05) are shown in boldface type.

### Central metabolism of *C. tyrobutyricum*.

**FIG 3:**
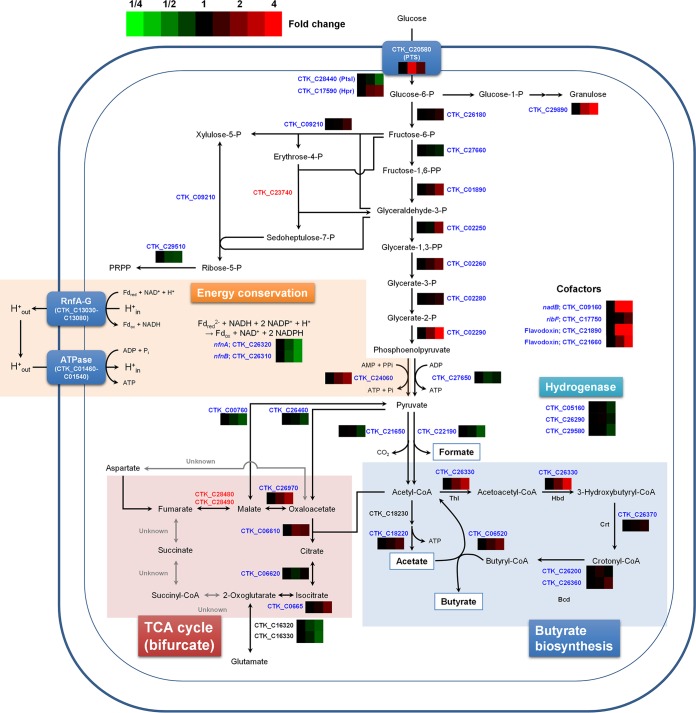
Protein changes of the central metabolic pathway in *C. tyrobutyricum*. The protein changes are from TMT-labeled identification results. Genes which products were identified are indicated in blue. Genes indicated in black are those genes whose products were not identified in TMT-labeled samples but were identified in label-free samples. Genes indicated in red are those genes whose products were not identified both in TMT-labeled and label-free peptide samples. Glucose-6-P, glucose-6-phosphate; Fd_ox_, oxidized ferredoxin.

An overview of the metabolic pathway in *C. tyrobutyricum* and changes in the expression levels of proteins involved in key metabolism are shown in Fig. 3. Shotgun proteomic data suggested that the phosphotransferase system (PTS) is mainly used in *C. tyrobutyricum* for glucose uptake. During the batch fermentation of *C. tyrobutyricum* on glucose, PTS enzyme I (CTK_C28440) and phosphocarrier protein Hpr (CTK_C17590) were identified at all phases. The results of genome sequence analysis suggest that there are two putative glucose-specific PTS enzyme II genes in *C. tyrobutyricum*: CTK_C27920 encoding only the IIA domain and CTK_C20580 encoding the entire set of IIA, IIB, and IIC domains. CTK_C27920 expression was not observed at any phase, while CTK_C20580 expression was observed in all three phases. Thus, the latter might be the major PTS II enzyme for glucose uptake. The expression levels of PTS II enzyme and HPr increased at C4 and C6, whereas PTS I decreased gradually.

Besides the PTS genes, three genes (CTK_C04350, -C07580, and -C21040) were predicted to encode sugar:H^+^ symporters, which might be used as alternative routes for glucose transport as in *C. beijerinckii* (29). However, only CTK_C04350 was weakly expressed at the stationary phase, suggesting that the PTS system described above is the major route for glucose transport in *C. tyrobutyricum*. In addition to glucose transporters, fructose-specific PTS (CTK_C06320; enzyme IIABC [EIIABC]) was abundantly expressed at the early exponential phase (C2), but its expression level decreased dramatically at the later phases (Table 3). This transient expression of the *fru* operon genes might be independent of catabolite repression in that the expression levels of catabolite control proteins, CcpA and histidine protein (HPr) kinase, were decreased at C6.

*C. tyrobutyricum* is found to catabolize glucose into pyruvate mainly via glycolysis. Genes related to the Entner-Doudoroff pathway and the oxidative branch of the pentose phosphate (PP) pathway are not present. Interestingly, a cluster (CTK_C23730 to -C23750) consisting of the *rpi* (ribose 5-phosphate isomerase), *tal* (transaldolase), and *tkt* (transketolase) genes was found in the genome, but none of their products were identified in the proteome. Additional *rpi* (CTK_C01400) and *tkt* (CTK_C09210) genes were found to be actively expressed, but no additional *tal* gene was found in the *C. tyrobutyricum* genome. As in other clostridial species, *C. tyrobutyricum* converts pyruvate into acetyl-CoA and CO_2_ using pyruvate:ferredoxin oxidoreductase. Of the two genes encoding pyruvate:ferredoxin oxidoreductase (CTK_C21650 and CTK_C25530), CTK_C21650 was constitutively expressed (20th, 24th, and 30th most abundant at C2, C4, and C6 phases, respectively), while CTK_C25530 was weakly expressed only at the C2 phase. Formic acid is produced by pyruvate:formate lyase (PFL); the genes encoding PFL (CTK_C22190) and PFL-activating enzyme (CTK_C22180) were expressed at all of the sampling points, but its expression level decreased as cells grew.

### Fermentative pathways in *C. tyrobutyricum*.

Previously, the *pta* (12), *ack* (11), and *ptb* (13) knockout mutant strains of *C. tyrobutyricum* ATCC 25755 have been reported. Based on the complete genome sequence of the KCTC 5387 (same as ATCC 25755) strain determined in this study, the fermentative pathways in *C. tyrobutyricum* were revisited. As expected, acetic acid is produced mainly through PTA and AK; the *pta* (CTK_C18230) and *ack* (CTK_C18220) genes are present in the *C. tyrobutyricum* genome. As in *C. acetobutylicum*, the expression of *pta* and *ack* was constitutive (30, 31). PTA was not identified after TMT labeling, but AK gradually increased from C2, reaching 1.5-fold increase at C6 (Fig. 3).

**FIG 4:**
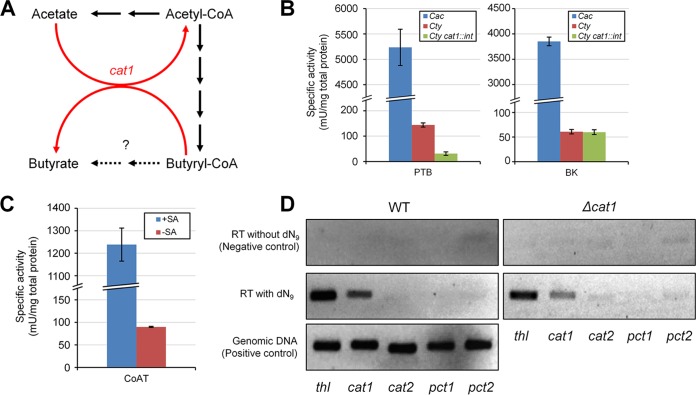
Butyric acid production in *C. tyrobutyricum* KCTC 5387 (ATCC 25755) depends on butyrate:acetate CoA transferase, not phosphotransbutyrylase (PTB) and butyrate kinase (BK). (A) Hypothesized pathway of butyric acid production in *C. tyrobutyricum* KCTC 5387. (B) Specific activities of PTB and BK in the crude extracts of *C. tyrobutyricum* (*Cty*) and *C. acetobutylicum* (*Cac*) ATCC 824. *C. acetobutylicum* was chosen as the positive control, as it has PTB and BK activities. (C) Specific activities of butyrate:acetate CoA transferase in the *C. tyrobutyricum* KCTC 5387 crude extract. The assay was performed in the presence (+) or absence (−) of sodium acetate (SA). (D) RT-PCR of four candidate CoA transferase genes in the wild-type (WT) and *cat1*::*int* strains of *C. tyrobutyricum*. The thiolase gene was chosen as the internal control.

Our biggest surprise was that the genes encoding PTB and BK are missing in the whole genome of the KCTC 5387 strain and in the draft genome sequences of the ATCC 25755 strain that were previously reported (21, 22). Consistent with this finding, our numerous attempts to amplify the partial *ptb* fragment according to the previous study (13) were not successful (see Fig. S4 in the supplemental material). This result suggests that the *ptb* and *buk* genes are indeed absent in the *C. tyrobutyricum* genome, and butyric acid is produced by *C. tyrobutyricum* using a pathway that is different from PTB-BK pathway used by *C. acetobutylicum* and *C. beijerinckii* (Fig. 4A). To further confirm this, the enzyme activities of PTB and BK were measured in the crude extracts of *C. tyrobutyricum* and *C. acetobutylicum* ATCC 824 (as a positive control). The crude extract of *C. tyrobutyricum* showed much lower activities of PTB and BK compared to those of *C. acetobutylicum* (Fig. 4B), strongly supporting the absence of PTB and BK in *C. tyrobutyricum*. The PTB and BK activities in the *C. tyrobutyricum* extract were 0.144 and 0.061 U/mg protein, which were only 2.7% and 1.6% of those in the *C. acetobutylicum* extract, respectively; the residual weak PTB/BK activities seem to come from the cross-activities of PTA/AK.

It was suggested that butyrate:acetate CoAT is required for butyrate biosynthesis in *C. kluyveri* (25). There are four genes encoding different CoA transferases in the *C. tyrobutyricum* genome. Products of two genes (CTK_C06520 and CTK_C07660) are homologous to *C. kluyveri* CoAT (25), and the other two genes (CTK_C16610 and CTK_C28060) share homology with the propionate:acetate CoAT from *C. propionicum* (32). There were no orthlogs of *C. acetobutylicum* CtfAB in *C. tyrobutyricum* as previously suggested (23). As the cell extract of *C. tyrobutyricum* clearly showed CoAT activity (Fig. 4C), we aimed to identify the key CoAT responsible for butyrate formation. Based on both reverse transcription-PCR (RT-PCR) (Fig. 4D) and proteome analyses, it was concluded that the *cat1* (CTK_C06520) gene is the only gene responsible for butyrate biosynthesis. To see whether CoAT encoded by the *cat1* gene is truly responsible for butyrate formation, this gene was knocked out using a mobile group II intron. However, insertion of a mobile group II intron into the coding sequence (CDS) in the sense direction (*cat1*::*intS* strain) did not decrease butyric acid production during batch fermentation (data not shown). RT-PCR of the *cat1*::*intS* strain suggested that the other putative CoAT genes were not turned on in consequence of *cat1* inactivation either (Fig. 4D); in fact, only the wild-type mRNA of the *cat1* gene was observed, suggesting that the active Cat1 was still produced after the resplicing of the mutant mRNA due to the presence of the knockout plasmid. To solve this problem, we tried plasmid curing in the *cat1*::*intS* strain and inserting the intron in the antisense direction, both of which were not successful. Insertion of the intron in the antisense direction into the *pta* and *ack* genes was also not successful; as well-known in the field, knocking out certain genes in clostridial strains can be extremely difficult, which was the case in this study.

### Energy conservation.

There are three energy conservation mechanisms found in the *C. tyrobutyricum* genome (Fig. 3): Rnf complex (CTK_C13030-CTK_C13080), NfnAB (CTK_C26320 and CTK_C26310), and pyruvate-phosphate dikinase (CTK_C24060). The Rnf complex generates NADH and proton gradient using reduced ferredoxin (25, 33). The proton gradient generated can be used to generate ATP via F_o_F_1_ ATPase. The NfnAB complex was first characterized in *C. kluyveri*, and it generates two NADPH molecules by the oxidation of one reduced ferredoxin and one NADH (34). Pyruvate-phosphate dikinase (PPDK) can generate ATP using phosphoenolpyruvate, AMP, and pyrophosphate (35).

Based on the proteomic analysis, the major energy conservation mechanisms when using glucose involve NfnAB and PPDK. The expression of the Rnf complex was apparently weak; only RnfC was identified in the label-free shotgun proteome. Both NfnA and NfnB were strongly expressed at early exponential phase; NfnA and NfnB were the 82nd and the 38th most abundant proteins, respectively, in the label-free proteome at C2. However, expression of NfnAB gradually decreased as cells grew; both NfnA and NfnB in the TMT-labeled proteome decreased by about 50% at C6 compared to C2. Expression of PPDK was not strong at C2 (the 741st most abundant); it was upregulated at C4 (1.6-fold compared to C2) and C6 (2.2-fold increase compared to C2).

### Sporulation.

Sporulation is a key characteristic of *Bacillus* and *Clostridium* species (30, 36). Even though *Clostridium* shares many proteins involved in the sporulation process with *Bacillus*, the mechanism of sporulation initiation in *Clostridium* is known to be different from that in *Bacillus*. The clostridial sporulation program has recently been reviewed thoroughly (36), but it might be different even among clostridial species. Since *C. tyrobutyricum* is able to undergo the sporulation process as well, the expression patterns of the sporulation genes were compared with those in other clostridial species.

Several genes for the initiation of sporulation were found in the genome, and the changes in the expression levels of some of these genes during the fermentation are shown in Fig. S3 in the supplemental material. The initiation of sporulation appears to be similar to those in other clostridial species. The expression level of Spo0A (CTK_C1943), the master regulator of sporulation, increased as fermentation proceeded. The expression level of AbrB protein (CTK_00640), which represses the *sigH* gene (CTK_29050) encoding σ^H^ and is repressed by phosphorylated Spo0A, decreased. This further promotes increased expression of Spo0A. It has been reported that orphan histidine kinases (HKs) directly phosphorylate Spo0A in clostridia (14, 37, 38). Two genes encoding putative orphan HKs (CTK_02350 and CTK_C04290) are present in the genome. Also, there is evidence in clostridial species that σ^K^ is involved at the early sporulation cascade (39, 40).

Once the forespore is developed, expression of the sporulation genes is spatially differentiated (see Fig. S3 in the supplemental material). While the early sporulation proteins could be identified in the proteome, those involved in the later stages seemed to be expressed rather weakly. It is known that in the forespore, *spoIIAA* (CTK_C08840), *spoIIAB* (CTK_C08850), and *spoIIE* (CTK_C29350) are involved in the control of σ^F^ (CTK_C08860) activity. The abundance of these proteins was much lower than that of Spo0A. Even though SpoIIAA and SpoIIE were not identified at some or all phases in nonlabeled experiments, it was possible to observe their relative changes after TMT tagging. SpoIIAB was induced at C4 and showed strongest expression at C6, whereas SpoIIE slightly decreased at C4 and increased at C6 compared to C2. This pattern is similar to previous cDNA microarray studies in *C. acetobutylicum* (31, 41). The expression levels of SpoIIAA and σ^F^ in the total protein pool were much lower than SpoIIAB (Fig. S3 in the supplemental material), which is unexpected as *spoIIAA*, *spoIIAB*, and *sigF* constitute an operon in *C. tyrobutyricum* as in other species. Both σ^E^ and σ^F^ were identified only at C6. Other key proteins including SpoIIGA (CTK_C18750), σ^G^ (CTK_C18730), and σ^K^ were not identified.

## DISCUSSION

*C. tyrobutyricum* is a potentially versatile microbial cell factory for the production of various chemicals due to its proven ability to produce butyric acid to a high titer with high-level product tolerance. It is however difficult to perform metabolic engineering of this strain due to the lack of understanding of its genotypic and metabolic characteristics at the genome level. This is why we decided to determine the complete genome sequence beyond an available draft genome sequence and perform genome-wide proteomic studies during the course of batch fermentation. One interesting feature of the *C. tyrobutyricum* genome is its compactness; its genome size is about 78% and 65% of that of its closest relatives *C. kluyveri* and *C. ljungdahlii*, respectively (Table 1). The *C. tyrobutyricum* genome contains 20 multiplicated genes encoding putative transposases, whereas at most, 5 genes are present in the *C. ljungdahlii* genome. This large number of insertion sequence (IS) elements might contribute to the loss of genes in *C. tyrobutyricum* via homologous recombination (42). A region with inverted GC skew located from 318° to 322° of the chromosome strongly supports our hypothesis (Fig. 1). This inverted region is flanked by two duplicated transposase genes (CTK_C27190 and CTK_C27510) oriented in the opposite direction, indicating that this inversion is due to homologous recombination.

From the whole-genome and shotgun proteome data, most genes involved in central metabolism could be identified. On the basis of the genome information, *C. tyrobutyricum* depends on the nonoxidative PP pathway to synthesize precursors of nucleotides and amino acids. However, transaldolase was not identified in the shotgun proteome; additional *rpi* and *tkt* genes, distantly located from the *rpi*-*tal*-*tkt* operon, were actively expressed (Fig. 3). It was suggested in *Clostridium stercorarium*, which does not have a transaldolase gene but utilizes xylose as the sole carbon source, that 3-deoxy-d-arabino-heptulosonate-7-phosphate synthase might replace the role of transaldolase (43). In *C. tyrobutyricum*, this gene (CTK_C27090) was strongly expressed (see Data Set S1 in the supplemental material), supporting this hypothesis.

As clostridial species do not have an oxidative phosphorylation mechanism, a major role of tricarboxylic acid (TCA) cycle might be the generation of precursors for various building blocks including amino acids. It is suggested that *C. acetobutylicum* uses a bifurcated TCA cycle (44, 45). Several key enzymes, including (Re)-citrate synthase, pyruvate carboxylase, NAD-dependent malic enzyme and malate dehydrogenase, were identified in the shotgun proteome of *C. tyrobutyricum*. Unlike *C. acetobutylicum*, however, α-ketoglutarate:ferredoxin oxidoreductase, which is required for the synthesis of succinyl-CoA from α-ketoglutarate, is absent in the *C. tyrobutyricum* genome and also in the genomes of *C. kluyveri* and *C. ljungdahlii*. Since these strains have only homoserine *O*-succinyltransferase, how succinyl-CoA is synthesized for methionine biosynthesis remains a question.

In many clostridial species, ferredoxin mediates the electron transfer of several enzymes including hydrogenase and pyruvate:ferredoxin oxidoreductase. Pyruvate:ferredoxin oxidoreductase uses ferredoxin as the major electron acceptor under high-iron conditions, but flavodoxin is an alternative electron acceptor when iron is limited (46). The expression of one flavodoxin protein (CTK_C2203) dramatically increased from the late exponential phase (ca. 10-fold); in fact, estimated abundance of the flavodoxin protein based on its NASF value indicated that this protein constituted ca. 3% of the total proteins identified. This result is in agreement with the previous findings using *Clostridium formicoaceticum* (46).

One of the most striking findings was that *C. tyrobutyricum* uses the CoAT pathway for butyric acid production, rather than the PTB-BK pathway. The strain employed for genome sequencing in this study was *C. tyrobutyricum* KCTC 5387, which is the same strain as ATCC 25755 that has been draft sequenced before. The partial sequence of the *pta* gene reported for ATCC 25755 (12) is almost identical to that of the KCTC 5387 strain (see Fig. S1 in the supplemental material for amino acid sequence alignment). In the case of *ack* (11), however, neither nucleotide nor amino acid sequence is similar to that of the KCTC 5387 strain (see Fig. S2 for amino acid sequence alignment). In fact, the partial AK sequence reported by X. Liu et al. (11) is exactly the same as that of the *Acinetobacter* sp. strain AK. Y. Zhang et al. (13) have reported inactivation of the *ptb* gene in *C. tyrobutyricum* ATCC 25755. However, there were neither *ptb* nor *buk* in the complete genome of the *C. tyrobutyricum* KCTC 5387 strain, which was further supported by *in vitro* enzyme assay (Fig. 4B). Just in case the KCTC 5387 strain deposited at the Korean Collection for Type Cultures (KCTC) is different from ATCC 25755, we ordered a new ATCC 25755 strain from ATCC and examined the newly obtained strain for the presence of *ptb*; the *ptb* fragment could be amplified neither from KCTC 5387 nor from ATCC 25755 and DSM 2637 (Fig. S4). Thus, the previous results reported on the presence and knockout of *ptb* (13) need to be validated.

Despite the absence of *ptb* and *buk*, *C. tyrobutyricum* is still capable of producing a large amount of butyric acid using butyrate:acetate CoAT (Fig. 4C; see Fig. S5 in the supplemental material). Based on our results of homology-based search, RT-PCR, and proteome analyses, the *cat1* gene was found to be responsible for this activity (Fig. 3 and 4D). We tried disrupting the *pta*, *ack*, and *cat1* genes using a mobile group II intron, but all of these attempts were not successful. On the basis of our hypothesis (Fig. 4A), elimination of acetate biosynthesis would lead to no fermentation end product; thus, the *pta* and *ack* genes are essential. It is known that electron transfer from NADH to ferredoxin is thermodynamically infeasible in clostridial species, and butyryl-CoA dehydrogenase bifurcates electrons from two NADHs to crotonyl-CoA and ferredoxin (47). Reduced or lack of butyric acid production seems to affect the electron bifurcation, which makes the cells unable to oxidize NADH; this is most likely why the *cat1* gene could not be knocked out. The ATP yield obtainable through the use of butyrate:acetate CoAT is equivalent to that obtainable with PTB-BK because butyrate formation by CoAT is coupled with acetate formation during which ATP is generated.

Endospore formation is one of the important characteristics of clostridia. For the initiation of sporulation, clostridial strains directly phosphorylate Spo0A by orphan HKs. As in other clostridial species, *spo0F* and *spo0B* are not present in the *C. tyrobutyricum* genome, suggesting that the Spo0A protein might be directly phosphorylated by orphan HKs. However, even though two genes encoding putative orphan HKs (HKs; CTK_02350 and CTK_C04290) are found in the *C. tyrobutyricum* genome, the products of these two genes were not identified in the proteome samples. The current clostridial sporulation model is based on the transcriptional changes, and our result does not necessarily suggest that *C. tyrobutyricum* has a different mechanism for sporulation initiation. This should be addressed in the future by construction of various mutant strains and comparison of transcriptional and translational level changes in these mutants.

In this paper, we reported the complete genome of *C. tyrobutyricum* KCTC 5387 (ATCC 25755). On the basis of genomic and accompanying proteomic analyses during the course of batch fermentation, we were able to provide new insights on its butyric acid production pathway, energy conservation mechanisms, sporulation processes, and other metabolic characteristics. The results and data presented in this paper will be valuable in better understanding the physiological characteristics of *C. tyrobutyricum* and developing metabolic engineering strategies for constructing high-performance strains capable of producing various chemicals of interest. As stated above, it will also be important to validate previous results of butyric acid production through the reported PTB-BK pathway (13), as our results suggest that this pathway does not exist in the same strain examined (KCTC 5387 or ATCC 25755); instead, butyric acid is produced through the CoAT pathway in *C. tyrobutyricum*.

## MATERIALS AND METHODS

### Batch fermentation of *C. tyrobutyricum*.

*C. tyrobutyricum* spores suspended in 2× YTG medium (glucose, 5 g liter^−1^; NaCl, 4 g liter^−1^; Bacto yeast extract, 10 g liter^−1^; Bacto tryptone, 16 g liter^−1^) supplemented with 15% (wt/vol) glycerol were inoculated in 10 ml of 2× YTG medium and heat shocked at 80°C for 10 min to kill the vegetative cells remaining in the suspension. After the heat shock, the test tube was incubated at 37°C in an anaerobic cabinet (Coy Laboratory Products, MI). A 500-ml capped flask containing 200 ml of the SP2 medium was inoculated with 5 ml of the 2× YTG culture grown overnight and incubated at 37°C until the OD_600_ reached to 1.0 to 2.0. This flask culture was used to inoculate a 5-liter bioreactor (LiFlus GX; Biotron, Gyeonggi-do, South Korea) containing 1.8 liters of SP2 medium. The SP2 medium contains the following ingredients (all per liter): glucose, 100 g; KH_2_PO_4_, 0.5 g; K_2_HPO_4_, 0.5 g; MgSO_4_·7H_2_O, 0.2 g; MnSO_4_·5H_2_O, 0.014 g; FeSO_4_·7H_2_O, 0.01 g; (NH_4_)_2_SO_4_, 2 g; Bacto yeast extract, 5 g; CH_3_COONa·3H_2_O, 5 g. During the fermentation, agitation speed and temperature were controlled at 200 rpm and 37°C, respectively. The pH of the fermentation medium was maintained at 6.0 by automatic addition of 7.5 M ammonia solution.

### Isolation of genomic DNA and genome sequencing.

For genomic DNA isolation, *C. tyrobutyricum* KCTC 5387 grown in 2× YTG medium was harvested at late exponential phase. Total DNA from *C. tyrobutyricum* KCTC 5387 used for the genome sequencing was extracted using a G-Spin total DNA extraction minikit (Intron Biotechnology, Seongnam, South Korea) according to the manufacturer’s protocol. The DNA prepared was fragmented to construct the library which was read using an Ion Torrent PGM 316 Chip sequencer at Genotech (Daejeon, South Korea). A total of 3,024,966 reads with the average length of ca. 230 bp were obtained and assembled into 96 contigs by a CLC Genomics Workbench 5.5.2 software (CLC Bio, Denmark). The maximum and average sizes of the contigs were ca. 190 kb and 31 kb, respectively. The complete genome sequence was assembled by comparing our contig sequences to other shotgun genome sequences of *C. tyrobutyricum* DSM 2637 and ATCC 25755 (21, 22) and performing PCR analysis. The ambiguous sequences compared to other draft genomes were further confirmed by PCR and Sanger sequencing.

### Sample preparation for proteome analysis.

*C. tyrobutyricum* cells (about 10 OD_600_/ml) were homogenized in 1 ml of lysis buffer (10 mM Tris-HCl [pH 8.0], 1.5 mM MgCl_2_, 10 mM KCl, 0.5 mM dithiothreitol [DTT], 0.5 mM Pefabloc SC, and 0.1% SDS) using Precellys 24 (Bertin Technologies, France) three times for 20 s each time at 6,500 rpm with a 5-min pause between homogenization cycles to prevent overheating. The supernatant was separated by centrifugation at 18,000 rpm for 30 min at 4°C, the protein concentration was determined using a Bradford protein assay (Bio-Rad), and aliquots were stored at −80°C. Each protein extract (100 µg) was reduced with 500 mM tris(2-carboxyethyl)phosphine (TCEP) at room temperature for 60 min and then alkylated with 500 mM iodoacetamide (IAA) at room temperature in the dark for 60 min. The samples were desalted using a membrane filter of 10 kDa cutoff and dissolved in 200 mM triethylammonium bicarbonate (TEAB) buffer to a final concentration of 1 µg/µl. Sequencing-grade trypsin (Promega, Madison, WI) was added at 1:20 (wt/wt) into proteins in TEAB buffer and incubated overnight at 37°C.

### TMT labeling.

Each peptide sample of individual proteins extracted from three C2, C4, and C6 sample points of *Escherichia coli* was divided into two fractions and was individually labeled using a TMTSixplex (TMT stands for tandem mass tag) reagents kit (catalog no. 90064, Thermo Scientific, MA) according to the manufacturer’s protocol. TMT-126 and TMT-129 were used to label C2, TMT-128 and TMT-131 were used to label C4, and TMT-127 and TMT-130 were used to label C6. Aqueous hydroxylamine solution (5%, wt/vol) was added to quench the reaction. The six samples were then combined, speed vacuum dried, and then dissolved in 50 µl of Milli-Q−water containing 0.1% formic acid for liquid chromatography coupled to tandem mass spectrometry (LC-MS/MS) analysis.

### 2D-LC-MS/MS analysis.

The TMT-labeled or label-free samples were analyzed using a two-dimensional LC-MS/MS (2D-LC-MS/MS) system consisting of a nanoACQUITY ultraperformance LC system (Waters, USA) and an LTQ Orbitrap Elite mass spectrometer (Thermo Scientific, USA) equipped with a nanoelectrospray source. The 2D-LC-MS/MS analysis was performed as previously reported by M. P. Washburn et al. (48) with slight modifications. Briefly, a strong cation exchange (SCX) (5 µm, 3 cm) column was placed just before the C_18_ trap column (inner diameter [i.d.], 180 µm; length, 20 mm; particle size, 5 µm; Waters). Peptide solutions were loaded in 5-µl aliquots for each run. Peptides were displaced from the SCX phase to the C_18_ phase by a salt gradient that was introduced through an autosampler loop and desalted for 10 min at a flow rate of 4 µl/min. The trapped peptides were then separated on a 200-mm homemade microcapillary column consisting of C_18_ (Aqua; particle size, 3 µm) packed into 100-µm silica tubing with an orifice i.d. of 5 µm. A 10-step salt gradient was performed using 3 µl of 0, 25, 50, 100, 250, and 500 mM ammonium acetate (0.1% formic acid in 5% acetonitrile [CAN]) and 4, 5, 9, and additional 9 µl and 500 mM CAN (0.1% formic acid in 30% ACN). The mobile phases A and B were composed of 0 and 100% acetonitrile, respectively, and each contained 0.1% formic acid. The LC gradient began with 5% mobile phase B for 1 min and was ramped to 20% mobile phase B over 5 min, to 50% mobile phase B over 80 min, to 95% mobile phase B over 4 min, and remained at 95% mobile phase B over 10 min and 5% mobile phase B for another 5 min. The column was reequilibrated with 5% mobile phase B for 15 min before the next run. The voltage applied to produce an electrospray was 2.0 kV. During the chromatographic separation, the LTQ Orbitrap Elite mass spectrometer was operated in a data-dependent mode. The MS data were acquired using the following parameters: five data-dependent collision-induced dissociation-high energy collision dissociation (CID-HCD) dual MS/MS scans per full scan; CID scans acquired in linear trap quadrupole (LTQ) with two-microscan averaging; full scans and HCD scans were acquired in Orbitrap at a resolution of 60,000 and 15,000, respectively, with two-microscan averaging; 35% normalized collision energy (NCE) in CID and 45% NCE in HCD; ±1-Da isolation window. Previously fragmented ions were excluded for 60 s. In CID-HCD dual scan, each selected parent ion was first fragmented by CID and then by HCD.

### Protein identification and quantification.

MS/MS spectra were analyzed using the following software analysis protocols with the protein sequence set of all *C. tyrobutyricum* proteins. The reversed sequences of all proteins were appended into the database for calculation of the false-discovery rate (FDR). ProLucid (49) was used to identify the peptides, with a precursor mass error of 25 ppm and a fragment ion mass error of 600 ppm. Trypsin was selected as the enzyme, with three potential missed cleavages. TMT modification (+ 229.1629) at the N terminus and lysine residue by the labeling reagent and carbamidomethylation at cysteine were chosen as static modifications. Oxidation at methionine was chosen as a variable modification. The CID and HCD tandem MS spectra from the same precursor ion are often combined by software to allow better peptide identification and quantification (50). We used a homemade software program where reporter ions from the HCD spectrum were inserted into the CID spectrum with the same precursor ion at the previous scan. Reporter ions were extracted from small windows (±20 ppm) around their expected *m*/*z* in the HCD spectrum. The output data files were filtered and sorted to compose the protein list using the DTASelect (51) with two and more peptide assignments for a protein identification and a false-positive rate of less than 0.01.

A quantitative analysis was conducted using Census in the IP2 pipeline (Integrated Proteomics, USA). The intensity at a reporter ion channel for a protein was calculated as the average of this reporter ion’s intensities from all constituent peptides from the identified protein (52). The measured intensity ratios of proteins were transformed to log_2_ scale. The resulting ratios were log transformed (base 2) in order to achieve a normal distribution.

In order to control quality in protein quantification, we labeled the same sample with two different TMT reagents. Since every protein was expected to have an abundance ratio of 1:1, in comparisons of different TMT-labeled samples for the same sample, data with more than 30% variation was excluded (for example, TMT-126 and TMT-129 for the C2 sample). TMT ratios for proteins were calculated over average reporter ion intensities over all peptides assigned to a protein subgroup. Proteins with *P* < 0.05 were defined as significantly changed in abundance. The false-discovery rate was performed by selecting a *P* value from the two biological replicates and ensuring that the fold change was significant in the other two conditions.

### Inactivation of the *cat1* gene using a mobile group II intron.

A retargeted intron fragment was amplified using primers in Table S1 in the supplemental material and cloned into pCACYS3 according to the previous studies (17, 53). After verification of the intron sequence, the retargeted pCACYS3 plasmid was digested with XmaI, and the 3.9-kb fragment containing the whole L1.LtrA cassette was subcloned into the XmaI-digested pMTL82251 plasmid. The resulting plasmid was transferred to *E. coli* CA434 (donor strain) and finally transferred to *C. tyrobutyricum* by conjugation according to the previous reports (15, 54). After 12-h mating, transconjugants were selected onto 2× YTG agar containing 240 µg ml^−1^ of d-cycloserine and 50 µg ml^−1^ of erythromycin. Transconjugant colonies were streaked again onto selective agar to exclude donor *E. coli* cells, and gene disruption was confirmed by colony PCR.

### Preparation of cell-free extracts.

Either *C. tyrobutyricum* or *C. acetobutylicum* cells were grown in 200 ml of the SP2 medium at 37°C. When OD_600_ reached about 1.0, the cells were harvested by centrifugation of 40 ml of the culture at 2,200 × *g* and 4°C for 15 min. The cells were washed once using distilled water, resuspended in 1 ml of 0.1 M potassium phosphate buffer solution (pH 7.4), and finally homogenized using a VC 750 sonicator (Sonics & Materials, CT). Cell debris was removed by centrifugation at 16,100 × *g* and 4°C for 10 min, and clear supernatants were used for enzyme assay. Total protein concentration was determined using the Bradford assay.

### Enzyme assay.

Phosphotransbutyrylase activity was determined by measuring liberation of free CoA from butyryl-CoA (55). Butyrate kinase activity was determined in the butyryl-phosphate-forming direction by measuring butyrohydroxamic acid (56, 57). CoAT activity was measured in the acetyl-CoA-forming direction by the method of W. Buckel et al. (58), using butyryl-CoA and acetate as the substrates; formation of acetyl-CoA was determined by liberation of free CoA by a coupled citrate synthase reaction (58). An Ultrospec 3100 Pro spectrophotometer (GE Healthcare, United Kingdom) was used for the enzyme assay. One unit of the enzyme was defined as conversion of 1 μM substrates per min.

### RNA isolation and RT-PCR.

For RNA isolation for reverse transcription-PCR (RT-PCR), all strains were cultivated in the anaerobic chamber at 37°C. An overnight 2× YTG culture of each *C. tyrobutyricum* strain was diluted 50 times into 200 ml of the SP2 medium (100 g liter^−1^ of initial glucose) contained in a 500-ml screw-cap flask and kept at 37°C until the OD_600_ reached 0.9 to 1.1. One milliliter of each SP2 culture was collected, and cells were harvested for 5 min at 4°C and 8,000 × *g*. RNA was isolated according to a hybrid protocol of an acid guanidinium thiocyanate-phenol-chloroform method using Easy-Red BYF (IntronBio, Gyeonggi-Do, South Korea) and a spin column purification method using RNeasy mini (Qiagen, Valencia, CA). Reverse transcription was performed using RocketScript RT premix (Bioneer, Daejeon, South Korea) according to the manufacturer’s protocol; 1 µg of total RNA and 100 pmol of random nonadeoxynucleotides (dN_9_) were subjected to a 20-µl RT reaction. When using gene-specific primers, 20 pmol of the reverse primer, which can bind to the target mRNA, was used instead of dN_9_. If required, DNase I was used to treat total RNA prior to RT reactions to destroy residual genomic DNA fragments. PCR was performed using the AccuPower PCR premix (Bioneer; 20-µl total reaction mixture volume). A T100 thermocycler (Bio-Rad, Hercules, CA) was used for all RT and PCRs throughout the study. The thiolase gene was chosen as an internal control of RT-PCR based on our shotgun proteome data (see Data Set S1 in the supplemental material). Primer sequences for PCR are described in Table S2 in the supplemental material.

### Nucleotide sequence accession numbers.

The genome sequence for *C. tyrobutyricum* has been deposited at GenBank under the following accession numbers: CP014170 (chromosome) and CP014171 (plasmid).

## SUPPLEMENTAL MATERIAL

Data Set S1 Proteins identified by label-free shotgun proteomics. Download Data Set S1, XLS file, 0.6 MB

Data Set S2 Protein changes determined by TMT isobaric tagging. Download Data Set S2, XLS file, 0.3 MB

Figure S1  Sequence alignment of the *C. tyrobutyricum* Pta protein. Download Figure S1, DOC file, 0.1 MB

Figure S2  Sequence alignment of the *C. tyrobutyricum* Ack protein. Download Figure S2, DOC file, 0.1 MB

Figure S3  Schematic diagram of the clostridial sporulation cascade and corresponding protein changes in *C. tyrobutyricum*. Download Figure S3, DOC file, 0.3 MB

Figure S4  Determination of the *ptb* gene in the *C. tyrobutyricum* genome. Download Figure S4, DOC file, 0.7 MB

Figure S5  Fed-batch fermentation profile of *C. tyrobutyricum* KCTC 5387. Download Figure S5, DOC file, 0.1 MB

Table S1  Primers for gene knockout experiments. Table S1, DOC file, 0.04 MB

Table S2  Primers for RT-PCR. Table S2, DOC file, 0.03 MB

Table S3  Primers for amplifying the *ptb* gene from *C. tyrobutyricum* genomic DNA. Table S3, DOC file, 0.03 MB
