# Supplementary material for: Deciphering Clostridium tyrobutyricum Metabolism Based on the Whole-Genome Sequence and Proteome Analyses
Source: mBio. 2016 Jun 14;7(3):e00743-16. doi: 10.1128/mBio.00743-16 (PMC4916380; doi:10.1128/mBio.00743-16)
Supplement: Figure S1 — Sequence alignment of the C. tyrobutyricum Pta protein. Download [file mbo003162838sf1.doc]

**FIG S1.** Sequence alignment of the *C. tyroburtyricum* PTA protein. Amino acid sequence of Pta in this study and the partial peptide sequence of AK (UniProt identifier: Q6PWX1) reported by Y. Zhu, et al. (1) were aligned using the BLOSUM62 matrix. Only identical and similar amino acids were shaded in color. Identical amino acids were indicated by black outline.

**References**
