# Supplementary material for: Deciphering Clostridium tyrobutyricum Metabolism Based on the Whole-Genome Sequence and Proteome Analyses
Source: mBio. 2016 Jun 14;7(3):e00743-16. doi: 10.1128/mBio.00743-16 (PMC4916380; doi:10.1128/mBio.00743-16)
Supplement: Figure S2 — Sequence alignment of the C. tyrobutyricum Ack protein. Download [file mbo003162838sf2.doc]

**FIG S2.** Sequence alignment of the *C. tyroburtyricum* AK protein. Amino acid sequence of AK in this study and the partial peptide sequence of AK (UniProt identifier: Q66SZ0) reported by X. Liu, et al. (1) were aligned using the BLOSUM62 matrix. Only identical and similar amino acids were shaded in color. Identical amino acids were indicated by black outline.

**References**
