# Supplementary material for: Deciphering Clostridium tyrobutyricum Metabolism Based on the Whole-Genome Sequence and Proteome Analyses
Source: mBio. 2016 Jun 14;7(3):e00743-16. doi: 10.1128/mBio.00743-16 (PMC4916380; doi:10.1128/mBio.00743-16)
Supplement: Figure S3 — Schematic diagram of the clostridial sporulation cascade and corresponding protein changes in C. tyrobutyricum. Download [file mbo003162838sf3.doc]

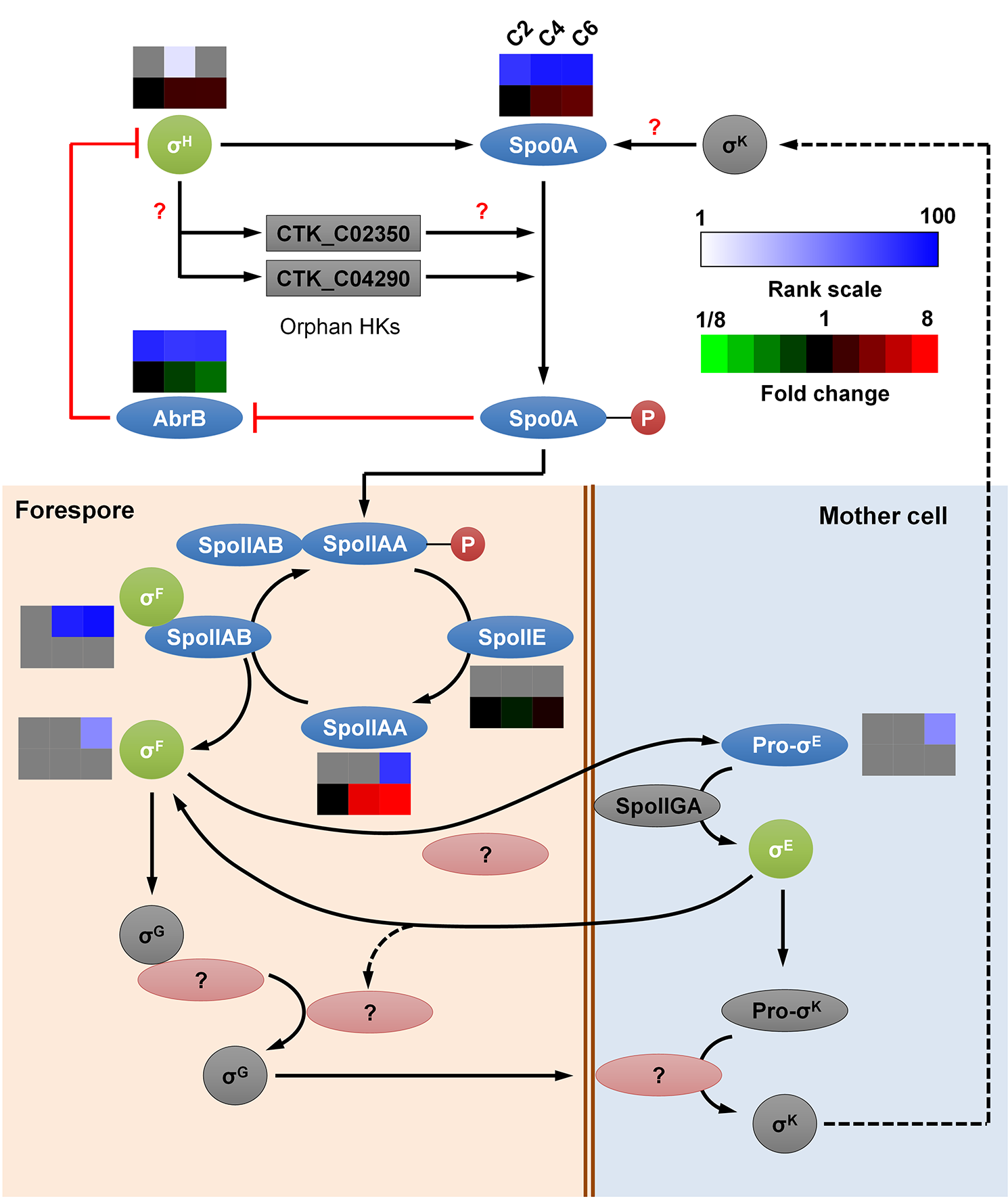


**FIG S3.** Schematic diagram of clostridial sporulation cascade and corresponding protein changes in *C. tyrobutyricum*. The rank of each protein in terms of abundance in the label-free peptide samples are shown in white-blue scale. The fold-change obtained from the TMT-labeled samples was also indicated below the rank scale. Proteins in grey items indicate that these proteins were identified neither in the TMT-labeled nor label-free samples.
