# Supplementary material for: Deciphering Clostridium tyrobutyricum Metabolism Based on the Whole-Genome Sequence and Proteome Analyses
Source: mBio. 2016 Jun 14;7(3):e00743-16. doi: 10.1128/mBio.00743-16 (PMC4916380; doi:10.1128/mBio.00743-16)
Supplement: Figure S4 — Determination of the ptb gene in the C. tyrobutyricum genome. Download [file mbo003162838sf4.doc]

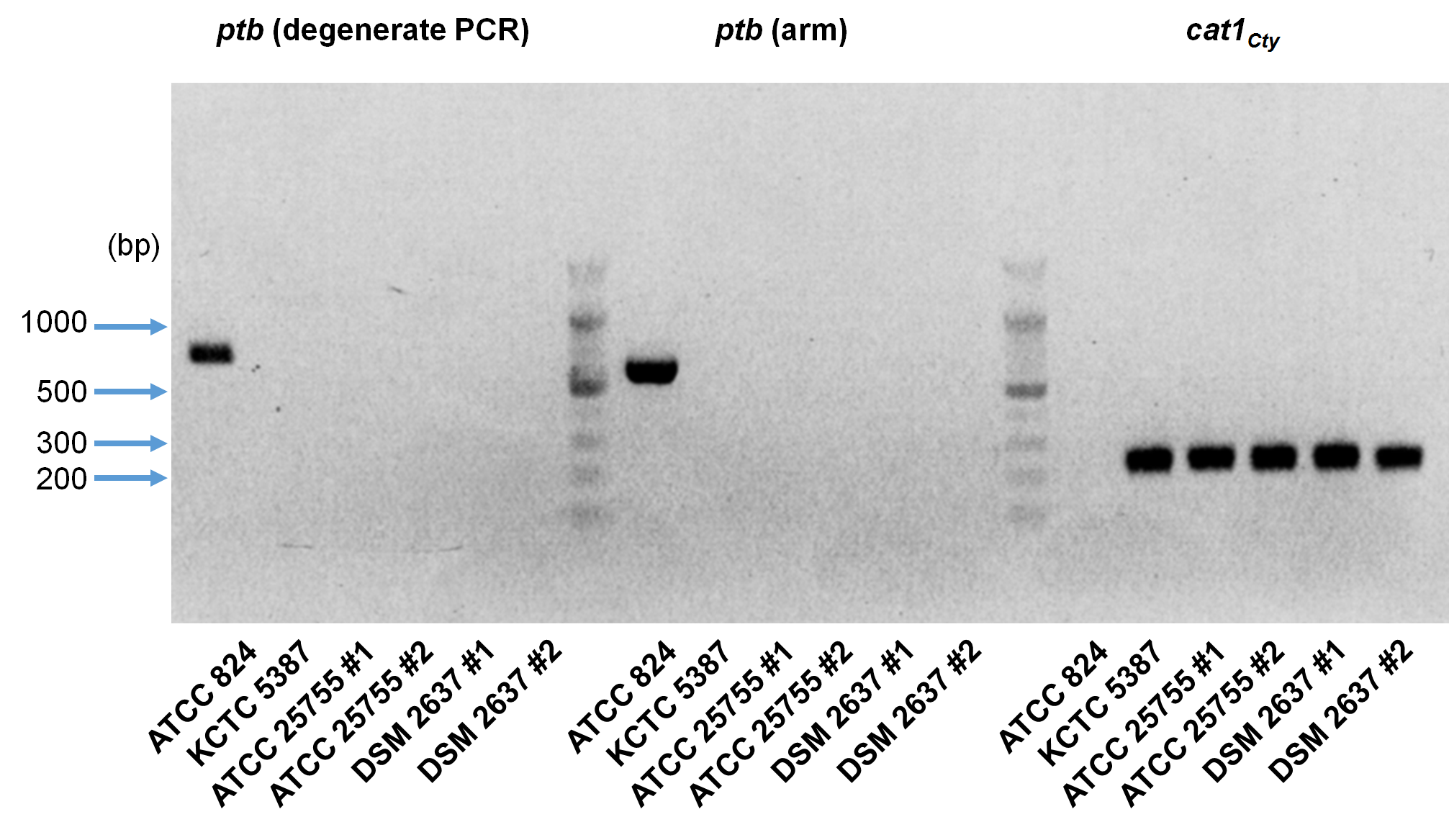


**FIG S4.** Determination of the *ptb* gene in the *C. tyrobutyricum* genome. Two primer sets, Ptb-degen and Ptb-KOarm (see Table S3) were used according to the previous study (1). The genomic DNA of *C. acetobutylicum* ATCC 824 was used as a control of *ptb*^+^ strain. Amplification of the *cat1* gene was performed as a control set (see Table S2). In case of the ATCC 25755 and DSM 2637 strains, the genomic DNA was directly isolated from the lyophilized stock (#1), and from the actively growing culture after inoculation (#2). For degenerate PCR, 5-cycle initial amplification was performed with lower annealing temperature (42°C) compared to the typical conditions, followed by a 35-cycle amplification with increased annealing temperature (52°C). For the other sets, PCR was performed using 40-cycle amplification with the annealing temperature of 55°C.

**Reference**
