# Supplementary material for: Deciphering Clostridium tyrobutyricum Metabolism Based on the Whole-Genome Sequence and Proteome Analyses
Source: mBio. 2016 Jun 14;7(3):e00743-16. doi: 10.1128/mBio.00743-16 (PMC4916380; doi:10.1128/mBio.00743-16)
Supplement: Figure S5 — Fed-batch fermentation profile of C. tyrobutyricum KCTC 5387. Download [file mbo003162838sf5.doc]

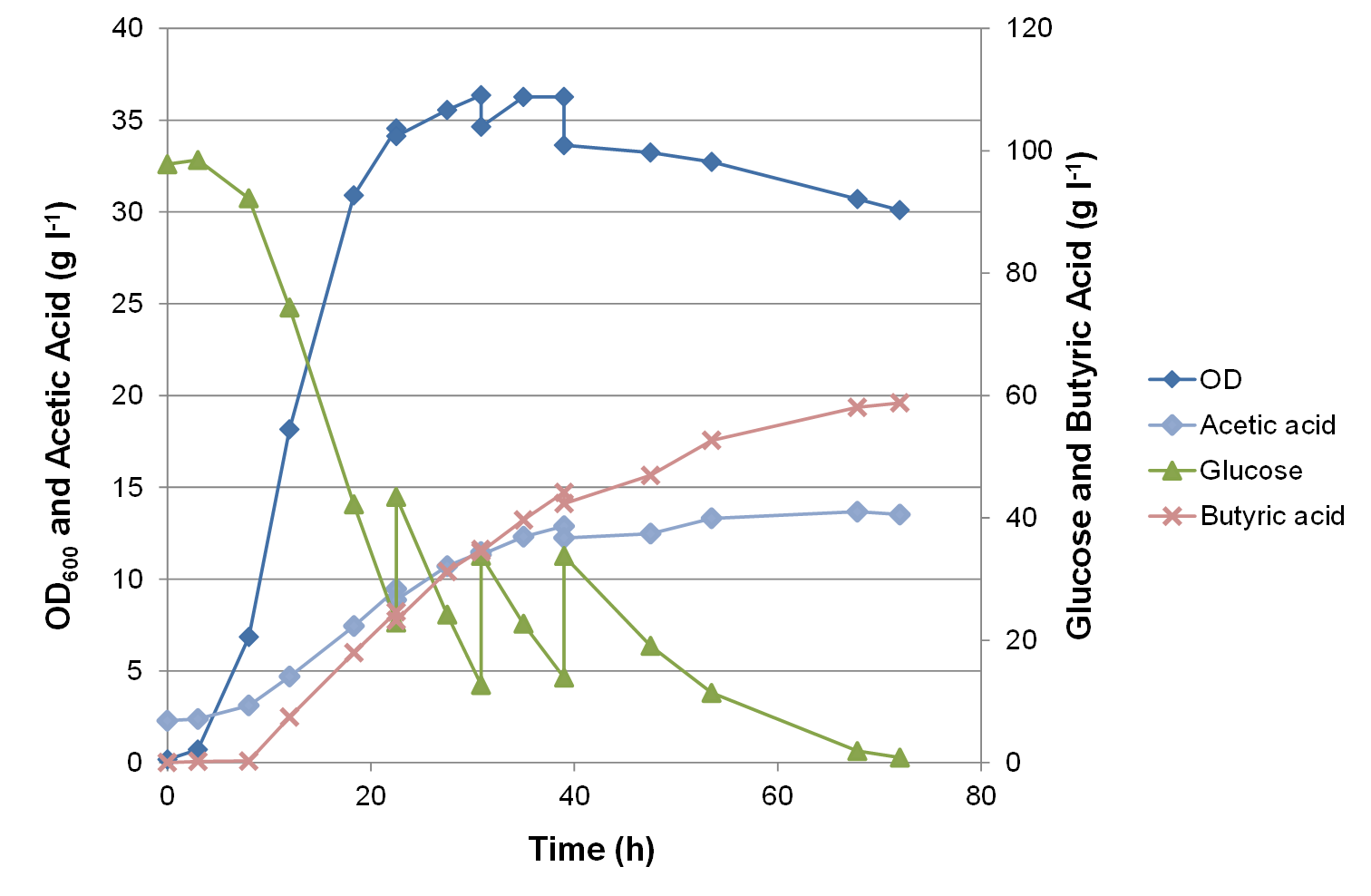


**FIG S5.** Fed-batch fermentation profile of *C. tyrobutyricum* KCTC 5387. Whenever the glucose concentration in the fermentation broth decreased below 25 g l^-1^, 100 ml of feeding solution containing 500 g l^-1^ glucose was added to the reactor. The other conditions were same as those of the batch fermentation described in Materials and Methods.
