# Supplementary material for: Deciphering Clostridium tyrobutyricum Metabolism Based on the Whole-Genome Sequence and Proteome Analyses
Source: mBio. 2016 Jun 14;7(3):e00743-16. doi: 10.1128/mBio.00743-16 (PMC4916380; doi:10.1128/mBio.00743-16)
Supplement: Table S1 — Primers for gene knockout experiments. [file mbo003162838st1.doc]

Table S1. Primers for gene knockout experiments

| Name | Sequence |
| --- | --- |
| EBS-Universal | CGAAATTAGAAACTTGCGTTCAGTAAAC |
| Cat1-410s-IBS | AAAAAAGCTTATAATTATCCTTAGTTTTCGAGTATGTGCGCCCAGATAGGGTG |
| Cat1-410s-EBS1d | CAGATTGTACAAATGTGGTGATAACAGATAAGTCGAGTATCATAACTTACCTTTCTTTGT |
| Cat1-410s-EBS2 | TGAACGCAAGTTTCTAATTTCGATTAAAACTCGATAGAGGAAAGTGTCT |
| Cat1-410s-colony-F | CAGAACCAGGAGTAGAAAAG |
| Cat1-410s-colony-R | CTGAATGTATTCCAAGGTCC |
| Cat1-362a-IBS | AAAAAAGCTTATAATTATCCTTATACTTCCTTCAAGTGCGCCCAGATAGGGTG |
| Cat1-362a-EBS1d | CAGATTGTACAAATGTGGTGATAACAGATAAGTCCTTCAATATAACTTACCTTTCTTTGT |
| Cat1-362a-EBS2 | TGAACGCAAGTTTCTAATTTCGGTTAAGTATCGATAGAGGAAAGTGTCT |
| Cat1-362a-colony-F | CGCAGGTACTTCTTAATGCT |
| Cat1-362a-colony-R | GAAGGCTGCATTTCAACAAC |
| Pta-429a-IBS | AAAAAAGCTTATAATTATCCTTAGAAGTCCCTGGAGTGCGCCCAGATAGGGTG |
| Pta-429a-EBS1d | CAGATTGTACAAATGTGGTGATAACAGATAAGTCCCTGGAGCTAACTTACCTTTCTTTGT |
| Pta-429a-EBS2 | TGAACGCAAGTTTCTAATTTCGATTACTTCTCGATAGAGGAAAGTGTCT |
| Pta-429a-colony-F | CTGAAGGCGAAGAAGAAAGA |
| Pta-429a-colony-R | ggtacaggatttacagcac |
| Ack-479a-IBS | AAAAAAGCTTATAATTATCCTTAGTACTCCATAAGGTGCGCCCAGATAGGGTG |
| Ack-479a-EBS1d | CAGATTGTACAAATGTGGTGATAACAGATAAGTCCATAAGTATAACTTACCTTTCTTTGT |
| Ack-479a-EBS2 | TGAACGCAAGTTTCTAATTTCGATTAGTACTCGATAGAGGAAAGTGTCT |
| Ack-479a-colony-F | ctgcagttggacatagagtt |
| Ack-479a-colony-R | tctagttcccatgcaaagac |
