# Supplementary material for: Deciphering Clostridium tyrobutyricum Metabolism Based on the Whole-Genome Sequence and Proteome Analyses
Source: mBio. 2016 Jun 14;7(3):e00743-16. doi: 10.1128/mBio.00743-16 (PMC4916380; doi:10.1128/mBio.00743-16)
Supplement: Table S2 — Primers for RT-PCR. [file mbo003162838st2.doc]

Table S2. Primers for RT-PCR

| Name | Sequence |
| --- | --- |
| Thl-F | TTGGATCCTAGCATAATGGG |
| Thl-R | GCTATTGCTCCTCCATTTAC |
| Cat1-F | GAAGATGGATCAACCCTACA |
| Cat1-R | CCCATTAGGAATGTGACAAC |
| Cat2-F | CCACTGGATCTTATTGATGC |
| Cat2-R | CTTCCAGATTTTACGGCTTC |
| Pct1-F | AGTAATAGGTGGACATTGGG |
| Pct1-R | TTTCCACCTTCAAGTCTAGG |
| Pct2-F | GGTTTTGTAGGCAGTCTTTG |
| Pct2-R | TAGGTGATAGATTCCAGTGC |
