# Supplementary material for: Deciphering Clostridium tyrobutyricum Metabolism Based on the Whole-Genome Sequence and Proteome Analyses
Source: mBio. 2016 Jun 14;7(3):e00743-16. doi: 10.1128/mBio.00743-16 (PMC4916380; doi:10.1128/mBio.00743-16)
Supplement: Table S3 — Primers for amplifying the ptb gene from C. tyrobutyricum genomic DNA. [file mbo003162838st3.doc]

Table S3. Primers for amplifying the *ptb* gene fragment from *C. tyrobutyricum* genomic DNA

| Name | Sequence |
| --- | --- |
| Ptb-degen-F | AATWRTAAAYGARCCTAACG |
| Ptb-degen-R | RCTRTCWGCTCTWGAAGTTA |
| Ptb-Harm-F | ACAACTGGTGCAGAAGTTCC |
| Ptb-Harm-R | GTAGAGCTTGTATCAACTGG |
